# Dissecting the expression of *EEF1A1/2* genes in human prostate cancer cells: the potential of EEF1A2 as a hallmark for prostate transformation and progression

**DOI:** 10.1038/bjc.2011.500

**Published:** 2011-11-17

**Authors:** B Scaggiante, B Dapas, S Bonin, M Grassi, C Zennaro, R Farra, L Cristiano, S Siracusano, F Zanconati, C Giansante, G Grassi

**Affiliations:** 1 Molecular Biology Section, Department of Life Sciences, University of Trieste, Via Giorgieri, 1, Trieste 34127, Italy; 2 University Department of Medical Sciences, Surgery and of Health, Hospital of Cattinara, Trieste, Italy; 3 Department of Chemical Engineering, University of Trieste, Trieste, Italy

**Keywords:** prostate cancer, EEF1A2, EEF1A1, LNCaP, PC-3, 22Rv1

## Abstract

**Background:**

In prostate adenocarcinoma, the dissection of the expression behaviour of the eukaryotic elongation factors (eEF1A1/2) has not yet fully elucidated.

**Methods:**

The EEF1A1/A2 expressions were investigated by real-time PCR, western blotting (cytoplasmic and cytoskeletal/nuclear-enriched fractions) and immunofluorescence in the androgen-responsive LNCaP and the non-responsive DU-145 and PC-3 cells, displaying a low, moderate and high aggressive phenotype, respectively. Targeted experiments were also conducted in the androgen-responsive 22Rv1, a cell line marking the progression towards androgen-refractory tumour. The non-tumourigenic prostate PZHPV-7 cell line was the control.

**Results:**

Compared with PZHPV-7, cancer cells showed no major variations in EEF1A1 mRNA; eEF1A1 protein increased only in cytoskeletal/nuclear fraction. On the contrary, a significant rise of EEF1A2 mRNA and protein were found, with the highest levels detected in LNCaP. Eukaryotic elongation factor 1A2 immunostaining confirmed the western blotting results. Pilot evaluation in archive prostate tissues showed the presence of EEF1A2 mRNA in near all neoplastic and perineoplastic but not in normal samples or in benign adenoma; in contrast, EEF1A1 mRNA was everywhere detectable.

**Conclusion:**

Eukaryotic elongation factor 1A2 switch-on, observed in cultured tumour prostate cells and in human prostate tumour samples, may represent a feature of prostate cancer; in contrast, a minor involvement is assigned to EEF1A1. These observations suggest to consider EEF1A2 as a marker for prostate cell transformation and/or possibly as a hallmark of cancer progression.

Prostate carcinoma is the main cause of cancer death in males in the Western world because switch from androgen-responsive to androgen-refractory phenotype typically occurs and the possibility of curative treatment is low. The molecular basis of tumour onset and progression has not yet fully elucidated.

Among the eukaryotic elongation factor 1A (eEF1A) family, two constitutive and actively transcribed genes are known: *EEF1A1* (6q14.1) and *EEF1A2* (20q13.3). Canonical functions of eEF1A proteins allow correct positioning of the aa-tRNA in the A site of the ribosome. In mammalian, eEF1A1 is ubiquitously expressed, whereas eEF1A2 expression is switched-on in adult life in specialised tissues such as skeletal muscle, cardio-myocytes and nervous system (Knudsen *et al*, 1993). Both are multifunctional proteins playing part in various important cellular mechanisms: eEF1A1 takes part in cytoskeletal remodelling, chaperone-like and proteosome-mediated protein degradation activities; it is also involved in the control of cell cycle, growth and death (Koiwai *et al*, 2008; Scaggiante *et al*, 2008; Kobayashi and Yonehara, 2009) and in the modulation of cell signalling (Yan *et al*, 2008); eEF1A2 participates in the activation of signalling pathways and modulation of cytoskeletal organisation (Panasyuk *et al*, 2008; Scaggiante *et al*, 2008; Kim *et al*, 2009).

Overexpression of EEF1A1 has been related to cell proliferation and cancer development in many tumours including head and neck, breast, leukaemia and hepatocarcinoma (Scaggiante *et al*, 2008). The expression of EEF1A2 in tissues other than specialised tissues has been associated to cancer development and aggressiveness, playing a role in ovarian, breast, lung, gastric, hepatic (Lee and Surh, 2009) and pancreatic (Cao *et al*, 2009) cancers.

Recently, Zhu *et al* (2009) have demonstrated that eEF1A is involved in the proliferation, invasion and migration of prostate cancer cells but no evidences for the differential role of eEF1A1 and eEF1A2 have been provided. To get more insight, we investigated the EEF1A1/A2 differential expression in a panel of prostate cancer cell lines: the androgen-responsive LNCaP and the non-responsive DU-145, PC-3 cells, described to display a low, moderate and high aggressive phenotype, respectively (Aalinkeel *et al*, 2004). Targeted experiments were also conducted in the 22Rv1 cell line marking the progression towards androgen-refractory tumour. Notably, this was done because ∼27 000 patients are expected to die of castration-resistant prostate cancer, the lethal form of the disease, in the United States in 2011 (http://www.cancer.gov). As control, the non-tumourigenic human prostate cell line, PZHPV-7 was considered. To ensure that the cells used mirror the above-described behaviours, their growth rate as well as the expressions of some cell cycle promoting genes such as E2F1-cyclin E1 were evaluated. E2F1 is a transcription factor directly implicated in the regulation of many cellular processes including cell proliferation. Upon retinoblastoma protein (pRb) phosphorylation by cyclin-dependent kinase (Cdk) bound to cyclin D, E2F1 is released from the pRb–E2F1 complex and induces the transcription of cyclin E1 which, bound to its Cdk, phosphorylates pRb and further increases the amount of free E2F1. Free E2F1 induces the transcription of many S-phase genes such as cyclin A (Attwooll *et al*, 2004).

Here, we focus on the expression of EEF1A1/A2 in a panel of human prostate cancer cells demonstrating that the switch-on of EEF1A2 gene expression associates with prostate cell transformation. Finally, pivotal evaluation of EEF1A1/A2 transcript presence in archive prostate tissues confirmed the observations in cultured cells.

## Materials and methods

### Cell lines and cultures

The adenocarcinoma prostate cell lines DU-145, PC-3, LNCaP, human colon adenocarcinoma LoVo DX and hepatocarcinoma HepG2 cells were cultured in 10% fetal bovine serum whereas the androgen-responsive 22Rv1 cell line (DSMZ, Braunschweig, Germany, ACC438) was grown in 20% fetal bovine serum containing 40% RPMI and 40% DMEM medium supplemented with 2 mM L-glutamine, 10 U ml^–1^ penicillin and 10 *μ*g ml^–1^ streptomycin (Euroclone, Milan, Italy). The non-tumourigenic prostate PZHPV-7 cell line was maintained in keratinocyte-serum-free medium (GIBCO-BRL, Invitrogen Corp., Carlsbad, CA, USA) containing 2 mM L-glutamine, 50 *μ*g ml^–1^ BPE, 5 ng ml^–1^ EGF (GIBCO-BRL, Invitrogen Corp.) and 1% antibiotics. Human lymphocytes from normal healthy donors were separated on Ficoll by standard procedure.

### MTT assay and S-phase cell evaluation

To evaluate cellular proliferation rate, the following seeding densities were set to assure optimal cell growth in 96-well microtiter plate: 2 × 10^3^ DU-145 and PC-3, 4 × 10^3^ LNCaP and 8 × 10^3^ PZHPV-7 in 200 *μ*l of the appropriate complete medium. Cell growth was evaluated by 3-(4,5-dimethylthiazol-2-yl)-2,5-diphenyl-tetrazolium bromide (MTT; Sigma, St Louis, MO, USA) according to the standard procedures. The evaluation of the cell number and the amount of S-phase cells were performed in six-well plates using the same seeding density adopted in 96-well microtiter plate (for 22Rv1, the same density of LNCaP was undertaken) using the double DNA labelling procedure as described (Baiz *et al*, 2009).

### RNA and DNA extraction

Total RNA and DNA were extracted from 2 × 10^6^ cells by using Tri-reagent procedure (Sigma Chem. Co.) and re-suspended in 50 *μ*l of DEPC water. The quality, integrity and quantification of total RNA were evaluated by spectrophotometric determination using a Bioanalyzer (Applied Biosystems, Foster City, CA, USA) and a NanoDrop ND-1000 (CelBio, Milan, Italy). The same procedure was followed to isolate total RNA from the fresh prostate benign adenoma sample, which was obtained from the Department of Urology, University of Trieste.

### cDNA synthesis and real-time PCR

In all, 2 *μ*g of total RNA was treated with amplification grade DNase I (Sigma Chem. Co.); afterwards reverse transcription was performed with random hexamer using MMV reverse transcriptase (Sigma Chem. Co.) following the producer's protocols.

The RT–PCRs were conducted in triplicate, utilising SYBRGreen Master Mix buffer (Applied Biosystems), 300 nM primers and 1 *μ*l of cDNA or 2 *μ*l of DNA (∼200 ng): 1 cycle at 95°C, 10 min; 40 cycles with a denaturing phase at 95°C, 15 s, an annealing phase at 62°C, 60 s and an elongation phase at 72°C, 30 s. A final extension at 72°C, 10 min and a dissociation stage (95°C/60°C/95°C for 15 s each) was then added. The relative amounts of the cDNA/DNA of target genes were normalised by 28S rRNA content according to Pfaffl (2004).

The sequences of the primers (Eurofins MWG Operon, Ebersberg, Germany) were EEF1A1cDNA fw 5′-AACATTGTCGTCATTGGACA-3′, EEF1A1cDNA rev 5′-ACTTGCTGGTCTCAAATTTC-3′ (NM_001402.4), which generated an amplification product of 229 bp; EEF1A2cDNA fw 5′-GCCACCGTCAATAGGTGGAC-3′, EEF1A2cDNA rev 5′-TGATGTGGGTCTTCTCCTTG-3′ (NM_001958.2) 183 bp; EEF1A1 DNA fw 5′-AACATTGTCGTCATTGGACA-3′ EEF1A1 DNA rev 5′-TTGATCTTTCCCTTTCTGGT-3′ (J04617), which generated an amplification product of 151 bp; EEF1A2 DNA fw 5′-ACCAAACATGGGGGCTTGGT-3′ EEF1A2DNA rev 5′-TCCTTGCCCATTTTGCTGGG-3′ (AF163763), which generated an amplification product of 206 bp. For 28S rRNA: fw 5′-TGGGAATGCAGCCCAAAG-3′, 28S rRNA rev 5′-CCTTACGGTACTTGTTGACTATGC-3′ (M11167.1), which generated an amplification product of 84 bp.

The specificity of the amplicons was previously demonstrated (Grassi *et al*, 2007). The copy number of EEF1A1/2 genes was calculated normalising by 28S ribosomal DNA and using the quantity of DNA of normal human peripheral blood lymphocytes as calibrator, assuming that these cells contain one copy of the genes for haploid genome.

### Western blotting

The proteins were extracted from 1 to 5 × 10^6^ cells: cytoplasmic proteins as described by Mansilla *et al* (2005) but stored in 10% glycerol, cytokeletal/nuclear-enriched proteins as previously described (Dapas *et al*, 2003). In all, 25 *μ*g of cytoplasmic or cytoskeletal/nuclear-enriched proteins were resolved onto 12% SDS–PAGE and blotted onto a 0.22-*μ*m nitrocellulose filter (Schleicher & Schuell, Keene, NH, USA). The following primary antibodies were used: eEF1A mouse monoclonal antibody (Upstate Biotechnology, Lake Placid, NY, USA), mouse polyclonal antibodies eEF1A1 and eEF1A2 all overnight at 4°C (Abnova Corporation, Taipei, Taiwan); rabbit polyclonal antibodies anti-cyclin D1, anti-cyclin A2 (Santa Cruz Biotechnology, Santa Cruz, CA, USA); mouse monoclonal antibodies anti-E2F1 and anti-cyclin E1, all 1 h at RT (BD Becton Dickinson Biosciences, San Jose, CA, USA). In the same filters, the loaded control proteins GAPDH (Santa Cruz) or *β*-actin (Sigma) were checked. Blots were developed using the corresponding secondary horseradish peroxidase antibodies (Santa Cruz) by enhanced chemilumiscence detection system (Pierce, Rockford, IL, USA) and exposed to Kodak film (Sigma).

### Immunofluorescence analysis

Prostate cell lines were plated on Thermanox plastic cover-slips (Nunc, Rochester, NY, USA), fixed with 1% paraformaldehyde in HBBS buffer (Hepes 10 mM, NaCl 150 mM, KCl 5 mM, CaCl_2_ 1.8 mM) for 30 min, RT and permeabilised with ice-cold methanol at 4°C, 2 min. After blocking in 0.5% bovine serum albumin, samples were incubated overnight at 4°C with anti-rabbit eEF1A2 polyclonal antibody (ProteinTech Group Inc., Chicago, IL, USA) rinsed with HBBS and incubated with an anti-rabbit IgG antibody conjugated to FITC (DAKO A/S, Glostrup, Denmark) for 1 h, RT. Images of immunostained cells were examined by Leica fluorescence microscope (Leica Microsystem, Wetzlar, Germany).

### RT–PCR on archive tissue samples

Finefix-fixed paraffin-embedded tissues of prostate cancers derived from four patients were kindly provided by Dr Ghimenton from the Surgical Pathology Unit of the Ospedale Civile Maggiore of Verona, Italy. The normal and perineoplastic tissues were analysed separately when encompassing >10% of the section area.

RNA was extracted from the paraffin-embedded dissected sections and treated with DNase as described elsewhere (Dotti *et al*, 2010). In all, 200 ng of RNA was retro-transcribed by MMV reverse transcriptase using random hexamer primers. For RT–PCR, the primers were those described in section ‘cDNA synthesis and real-time PCR’. Pre-denaturation was performed for 2 min at 95°C, followed by 30 cycles with a denaturation step of 30 s at 94°C, an annealing step of 30 s at 60°C and an extension step of 30 s at 72°C for EEF1A1 and 28S rRNA. For EEF1A2, the cycles were elevated at 50 and the annealing was done at 58°C. As negative controls paraffin blocks without tissues were used. The RNA quality in each clinical specimen was analysed by RT–PCR of 28S rRNA using 25 ng of total RNA and 35 cycles of amplification.

The nine prostate cancer and two prostate hyperplasia samples fixed in formalin and embedded in paraffin were obtained from the Pathology Department of the University of Trieste. Patients showed a median age at diagnosis of 63 years (25th–75th percentile=60–71). RNA was extracted according to Stanta and Schneider (1991) and treated with DNase. In all, 200 ng of RNA was transcribed using AMV reverse transcriptase with specific reverse primers. The RT–PCR was performed as previously reported (Stanta and Bonin, 1998), by using for EEF1A2 the following primers: Fw 5′-GCCACCGTCAATAGGTGGAC-3′ and Rv 5′-GATTCCGGAGCCGAGGTCTCA-3′ (*NM_001958)* giving a specific amplicon of 91 bp. In the cases (four) where the perineoplastic tissue was over 10% of the section, the perineoplastic and tumour tissue were analysed separately. Negative controls were paraffin blocks without tissues. The RNA quality in each clinical specimen was analysed by RT–PCR of 28S rRNA using 25 ng of total RNA and 35 amplification cycles. Amplification products were checked by dot blot or Southern blot hybridisation (Stanta and Bonin, 1998) using specific probes: 5′-CCCTCCCGGAGATAAAACCGCCGG-3′ for EEF1A2 and 5′-CGGGTGGTAAACTCCATCTAAGGC-3′ for 28S rRNA.

### Statistical analysis

Values were expressed as mean±s.e.m. Statistical significance was determined by one-way analysis of variance and the appropriate Student's test; a value of *P*<0.05 was considered to be statistically significant.

## Results

### Characterisation of human prostate cell lines proliferation behaviours

**Figure 1:**
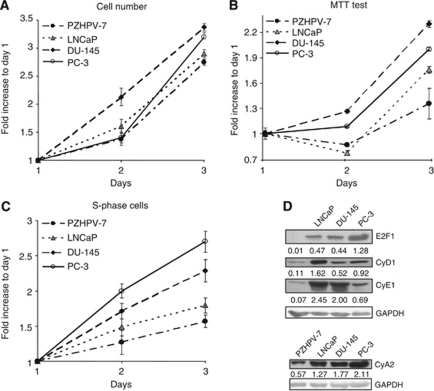
Cell proliferation and levels of some cell-cycle modulators. (**A**) *Cell numbers*. Cells were seeded in six-well plates and their numbers were evaluated at the indicated times. (**B**) *MTT test*. Cells were seeded in 96-well microtiter plates and the growth rate was measured at the indicated times by MTT incorporation. (**C**) *S-phase cells.* Cells were seeded in six-well plates and the amount of S-phase cell was evaluated at the indicated times. (**A**–**C**) The date are reported as fold increase respect to day 1; data are indicated as mean±s.e.m.; *n*=3. (**D**) *Western blotting of cell-cycle modulators*. Cells were collected at subconfluence; the levels of the indicated proteins are shown; GAPDH protein content was used as loading control. Relative quantifications are reported below.

The growth rates of the cancer LNCaP, DU-145, PC-3 and of the non-tumourigenic PZHPV-7 cell lines were assessed. As illustrated in Figure 1A–C, the three cancer lines showed a higher rate of proliferation compared with the control PZHPV-7. Notably, the LNCaP exhibited an evident lower rate of proliferation with respect to the less differentiated and more aggressive DU-145 and PC-3. The differences can be substantiated by cell counting (Figure 1A), MTT test (Figure 1B) and by the amount of S-phase cell (Figure 1C).

The increased proliferation rate of the three prostate cancer cells was paralleled by augmented protein levels of the pro-proliferative gene products E2F1, cyclin D1, cyclin E1 and cyclin A2, compared with the non-tumourigenic PZHPV-7 line (Figure 1D); increased expression of these genes typically associate with cancer transformation (Hwang and Clurman, 2005; Ladu *et al*, 2008; Li *et al*, 2010).

Within the three cancer cell lines, the LNCaP displayed the highest expression of cyclin D1, in agreement with its critical role in the regulation of cell-cycle progression in these cells (Comstock *et al*, 2007) and the highest level of cyclin E1 that may be related to the role of cyclin E1 as co-activator of androgen receptor (Cifuentes *et al*, 2003). In the less differentiated and more aggressive PC-3, we observed the highest levels of E2F1, typical of aggressive cancer phenotype (Ladu *et al*, 2008), and of its transcriptional-regulated target cyclin A2. This suggests that cyclin A2, could play an important role in prostate aggressiveness as demonstrated for breast cancer cells (Li *et al*, 2010).

Together these data support the concept of an increased proliferation attitude for the three cancer cell lines used compared with the non-tumourigenic control PZHPV-7 and the increased phenotypic aggressiveness for the DU-145 and PC-3 cells compared with the LNCaP ones.

### EEF1A1/A2 mRNA abundance and gene amplification in human prostate cell lines

**Figure 2:**
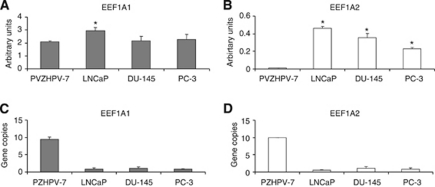
EEF1A1/2 mRNA levels and of EEF1A1/2 gene copies. (**A** and **B**) *Quantification of mRNA levels.* The EEF1A1/A2 mRNA levels evaluated in the indicated cell lines (normalised to 28S rRNA) are expressed as mean values±s.e.m., *n*=9. ^*^Marks the statistical significance with respect to PZHPV-7. (**C** and **D**) *EEF1A1/2 gene copies*. Gene copy number was calculated using normal lymphocyte DNA as calibrator. The results are expressed as mean values±s.e.m., *n*=3.

In the prostate cell lines, EEF1A1/2 mRNA levels were quantified by real-time PCR. To exclude amplification of the EEF1A1/2 retropseudogenes, DNase treatment of the samples was performed before retro-transcription reactions. With respect to PZHPV-7, no significant differences in EEF1A1 expression was found in DU-145 and PC-3 cell lines; a slight increase was observed in LNCaP (Figure 2A; *P*=0.0019). On the contrary, compared with PZHPV-7, a significant and remarkable rise of EEF1A2 mRNA levels was found in all three cancer cell lines with the highest level found in the LNCaP (Figure 2B; *P*<0.0001). To further verify whether the switch-on of EEF1A2 mRNA levels is a common feature of prostate carcinoma cell lines, measurements were conducted in another androgen-responsive cell line, that is, the 22Rv1, a typical form of tumour progression characterised by an androgen-refractory behaviour (Miyamoto *et al*, 2004). Also in the 22Rv1 cells, characterised by a growth rate comparable to LNCaP (see Supplementary material 1), the EEF1A1 mRNA levels did not significantly differ from those of PZHPV-7, whereas a significant (*P*=0.02) increase of EEF1A2 mRNA levels were found as in the other tumour cell lines tested (see Supplementary material 2A).

To understand whether the increment in EEF1A2 expression could be driven by gene amplification, primer pairs spanning unique regions in the two genes were used. Unexpectedly, only PZHPV-7 showed gene amplification: about nine copies of EEF1A1 and eight copies of EEF1A2 (Figure 2C and D).

### Abundance of eEF1A proteins in human prostate cell lines

The abundance of eEF1A1 and eEF1A2 were studied, by means of specific antibodies, in the cytoplasmic and in cytoskeletal/nuclear-enriched fractions. This latter because eEF1A proteins bind to actin modulating microtubule bundling and severing and this play a key role in invasion (Gross and Kinzy, 2005). The monoclonal antibody recognising both eEF1A1 and eEF1A2 forms was included as control for the total eEF1A proteins expression in the different compartments.

**Figure 3:**
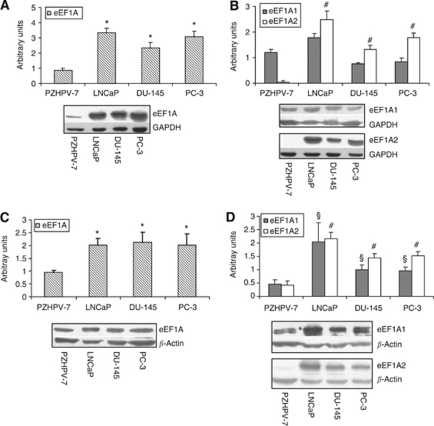
Quantification of total eEF1As and of eEF1A1/2 proteins in cytoplasmic and cytoskeletal/nuclear-enriched fractions. (**A**, **B**) Quantification of total eEF1As and eEF1A1/2 proteins in cytoplasmic extract. The blots were treated by either eEF1A (**A**) or eEF1A1/eEF1A2 antibodies (**B**). The quantifications are expressed as mean values±s.e.m., *n*=6 for eEF1A and *n*=3–10 for eEF1A1 and eEF1A2. The asterisks mark statistical significance: ^*^eEF1A and ^#^eEF1A2 with respect to PZHPV-7. Representative blots are shown below the graphs. (**C** and **D**) Quantification of total eEF1As and eEF1A1/2 proteins in cytoskeletal/nuclear extract. The blots were treated by either eEF1A (**C**) or eEF1A1/eEF1A2 antibodies (**D**). The quantifications are expressed as mean values±s.e.m., *n*=4 for eEF1A and *n*=3–8 for eEF1A1 and eEF1A2. The significance is indicated by ^*^eEF1A, ^§^eEF1A1 and ^#^eEF1A2 with respect to PZHPV-7. Representative blots are shown below the graphs.

In the cytoplasmic fraction, total eEF1A proteins was found to be increased in all tumour cells compared with PZHPV-7 (LNCaP, *P*=0.00005; DU-145, *P*=0.004; PC-3, *P*=0.001) (Figure 3A). This seems attributable to a significant rise of eEF1A2 but not of eEF1A1 (Figure 3B). In particular, eEF1A2 levels resulted significantly increased in all prostate cancer cell lines with the highest levels detected in LNCaP (LNCaP, *P*=0.000005; DU-145, *P*=0.000001; and PC-3, *P*=0.0000007 *vs* PZHPV-7). The eEF1A2 protein level was also consistently elevated in 22Rv1 (Supplementary material 2B), in agreement with the mRNA data.

In the cytoskeletal/nuclear-enriched fraction, we observed a significant increase of the total eEF1A protein levels in all tumour cells (LNCaP, *P*=0.02; DU-145, *P*=0.05; PC-3, *P*=0.04) compared with PZHPV-7 (Figure 3C). This was due to significant increase of both eEF1A1 (LNCaP, *P*=0.04; DU-145, *P*=0.05; PC-3, *P*=0.04) and eEF1A2 (LNCaP, *P*=0.006; DU-145, *P*=0.01; PC-3, *P*=0.001) (Figure 3D). Once again these observations were confirmed in the additional prostate tumour cell tested, that is, 22Rv1 (Supplementary material 2B).

### Immunofluorescence of eEF1A2 in human prostate cell lines

To confirm the intracellular presence and distribution of eEF1A2, immunofluorescence (IF) was performed. The eEF1A1 protein was not investigated due to the lack of an adequate commercial antibody for IF. The IF was performed in parallel for all cell lines, using the same fixing method, to properly compare the samples.

**Figure 4:**
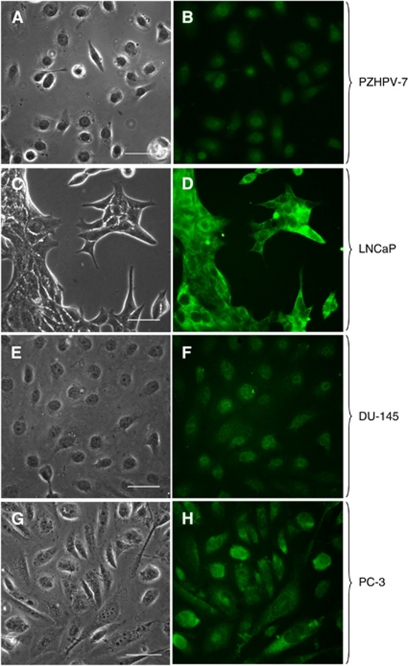
eEF1A2 IF. The exponentially growing cell lines indicated were plated on plastic cover-slips and fixed before the overnight incubation with eEF1A2 polyclonal antibody whose was revealed by an anti-rabbit IgG conjugated with FITC to fluorescence examination as described in Materials and Methods. (**A**, **C**, **E** and **G**) Contrast microscopy (the bar marks 50 *μ*M); (**B**, **D**, **F** and **H**) fluorescence microscopy.

The PZHPV-7 showed the global faintest fluorescence (mainly localised at nuclear level) with respect to the cancer cells, in agreement with the western blotting data (Figure 4B). The LNCaP showed the most intense fluorescence emission, well-matching the highest protein levels of eEF1A2 detected by western blotting. Notably, eEF1A2 was found to be mainly diffuse in the cytoplasm with minimal amounts in the nucleus (Figure 4D). This suggests that eEF1A2 found by western blotting in cytoskeletal/nuclear fraction (Figure 3D) is almost exclusively confined to the cytoskeleton. The DU-145 showed a broad distribution of the protein within the cells, with a more intense localisation in the nuclear region (Figure 4F). Finally, in the PC-3 cells, the fluorescence was present in both the cytoplasm and nucleus with some cells showing a discrete cytoplasmic and perinuclear localisation (Figure 4H). Notably, both DU-145 and PC-3 displayed lower eEF1A2 IF intensity than LNCaP, in agreement with western blotting results.

### EEF1A2 gene expression in human paraffin-embedded tissue samples

**Figure 5:**
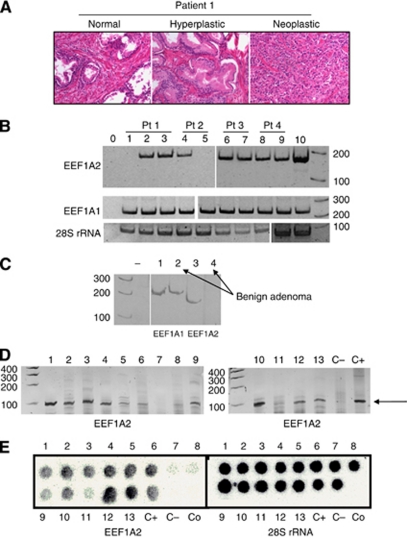
RT–PCR of EEF1A1/2 in human biopsy samples. (**A**) Dissection of Finefix-fixed paraffin-embedded samples. Prostate tissues histologic haematoxylin–eosin-stained sections of tissues from patient 1 are illustrated as an example ( × 20 magnification): normal tissue, hyperplastic peritumoural tissue, neoplastic tissue. (**B**) RT–PCR of Finefix-fixed paraffin-embedded samples. (1) Normal tissue, (2) hyperplatic peritumoural tissue, (3) neoplastic tissue from patient 1, (4) hyperplastic peritumoural tissue, (5) neoplastic tissue from patient 2, (6) hyperplastic peritumoural tissue, (7) neoplastic tissue from patient 3, (8) hyperplastic peritumoural tissue, (9) neoplastic tissue from patient 4 and (10) LoVoDX-positive control. (**C**) RT–PCR of human benign adenoma. The cDNA of fresh benign adenoma was amplified by using EEF1A1 primer pair giving amplicon of 229 bp (lanes 1 and 2) or EEF1A2 primer pair giving amplicon of 183 bp (lanes 3 and 4); lanes 2 and 4, adenoma; lanes 1 and 3, HepG2-positive control. (**D**) EEF1A2 RT–PCR on tissue archive formalin-fixed paraffin-embedded tissues. The samples were amplified with primer pair giving an amplicon of 91 bp. Lanes 1, 2, 4, 5, 7, 8, 9, 10 and 12 cancer samples; lanes 3, 6, 11 and 13 perineoplastic tissues; lane C+ positive control HepG2 cells; C− benign hyperplasia. The arrows mark the specific amplicon. (**E**) Probing of the amplicons on archive formalin-fixed paraffin-embedded tissues. The RT–PCR products shown in (**B**) were used to perform dot blotting with the specific EEF1A2 or 28S rRNA probes. Lanes 1, 2, 4, 5, 7, 8, 9, 10 and 12 prostate cancer samples; lanes 3, 6, 11 and 13, perineoplastic tissues; C− benign hyperplasia; C+ positive control HepG2, Co control of RT–PCR paraffin block.

The expression of EEF1A2 and EEF1A1 genes were analysed in four Finefix-fixed paraffin-embedded samples referred to four patients with a clinical diagnosis of prostate adenocarcinoma. This alcoholic fixation improved nucleic acids preservation in comparison with formalin (Dotti *et al*, 2010) and thus longer region could be amplified. For patient 1, it was possible to dissect the normal, peritumoural hyperplasia and neoplastic tissues (Figure 5A and B); for the other three patients, only peritumoural hyperplasia and neoplastic sections were dissectable. Three out of four tumour and all peritumoural hyperplasia samples resulted positive for EEF1A2 gene expression. On the contrary, the sample derived from normal tissue of patient 1 was negative for EEF1A2 mRNA (Figure 5B). All samples resulted positive for EEF1A1 expression, in agreement with the data obtained in cultured cells (Figure 2A). The quality of the RNAs obtained from each sample was proven by amplification of the 28S rRNA. Notably, in a fresh prostate benign adenoma sample (Figure 5C), EEF1A1 but not EEF1A2 expression was detected, further stressing the concept that EEF1A2 switch-on occurs in neoplastic tissue. The EEF1A2 analysis was then extended to formalin-fixed samples using primers pair amplifying a shorter region (91 bp, near the 5′-end of the transcript) than that used for the Finefix-fixed samples; the choice of a shorter PCR product was necessary due to the poor preservation of the nucleic acids in the formalin-fixed samples. The results in Figure 5D and E demonstrated the presence of the specific amplicon in seven out of nine tumour samples and in three out of four perineoplastic tissue. The specificity of the amplicon was confirmed by dot blotting using the specific probe for EEF1A2 amplicon; the quality of RT–PCR was confirmed by 28S rRNA amplification and probing (Figure 5D and E).

## Discussion

The involvement of eEF1A in prostate cancer biology has been recently proposed (Zhu *et al*, 2009). Here, we dissect the contribution of the two constitutive forms, that is, eEF1A1 and eEF1A2 in prostate cancer. The growing capacity and the expression of some cell-cycle-related genes were checked in the cell lines used, confirming the tumourigenic aggressiveness of the prostate cancer cell lines in the order PC-3⩾DU-145>LNCaP, with the PZHPV-7 behaving like non-tumourigenic cells (Figure 1) and the 22Rv1 similarly to LNCaP (see Supplementary material 1).

In general, an increased expression of eEF1As is thought to be related with tumourigenesis because rapidly proliferating cells need a higher protein synthesis activity. In all tumour cell lines, we found a strike increase in the total eEF1A proteins (Figure 3A and C), in good agreement with other data (Zhu *et al*, 2009). However, the most evident result of our investigation is the dramatic up-regulation of the expression of EEF1A2, but not of EEF1A1, in the prostate cancer lines at the mRNA and protein levels, compared with the non-tumourigenic prostate PZHPV-7 cells (Figures 2A and B, 3B and D; Supplementary material 2). Curiously, in the non-tumourigenic PZHPV-7, amplification of EEF1A1 and EEF1A2 genes was detected, even though these cells that barely express EEF1A2 and EEF1A1 expression did not result to be higher than in cancer cells. This might be the consequence of cell immortalisation by papilloma virus as demonstrated in non-cancerous cells of the cervix infected by HPV (Golijow *et al*, 2001).

The overexpression of EEF1A2 gene in cancer cells merely resulted from the switch-on of the gene without a significant gene amplification (Figure 2D) likely indicating that the activation and modulation of EEF1A2 transcription is the most remarkable event in prostate cancer cells. Notably, compared with PZHPV-7, in all cancer cells, eEF1A2 protein was significantly increased in both cytoplasmic and cytoskeletal/nuclear fractions (Figure 3B–D; Supplementary material 2B). This may suggest a role for eEF1A2 in sustaining both protein translation (cytoplasmic fraction) and cytoskeletal reorganisation (cytoskeletal–nuclear fraction) in the tumour cells studied. Worth of mark is the statistically significant lower levels of EEF1A2 expression in DU-145, PC-3 and 22Rv1 cells compared with LNCaP. Notably, representing 22Rv1, DU-145 and PC-3 cell lines different stages of tumour progression, it is not excluded that lower levels of eEF1A2 could be predictable of the outcome as found for breast tumours (Kulkarni *et al*, 2007). Additionally, the high levels of eEF1A2 expression detected in LNCaP does not seem to be a typical feature of androgen-responsive cells as in 22Rv1, the additional androgen-responsive cell line tested, EEF1A2 levels are comparable to DU-145 and PC-3 cells (Supplementary material 2). In contrast to eEF1A2, the cytoplasmic eEF1A1 did not significantly differ between non-tumourigenic and tumourigenic cells and an increase of the protein was seen only in the cytoskeletal/nuclear fraction but without significant variation between the cancer cell lines tested (Figure 3B). Thus, it is tempting to speculate that eEF1A1 generally contributes to prostate tumour onset by means of its moonlighting functions, that is, cytoskeleton modulation, and possibly genes expression, but it unlikely signs cancer progression.

The IF data on eEF1A2 confirm the western blotting results (Figure 3B and D), indicating the most intense fluorescence in LNCaP followed by those of DU-145 and PC-3 and the lowest in PZHPV-7 (Figure 4). In DU-145 and PC-3 cells, the fluorescence was mainly confined to the cytoplasm/cytoskeleton, whereas in the LNCaP cells, it was detectable also at nuclear/perinuclear level. These observations are in agreement with the findings of Kim *et al* (2009) of a redistribution of the eEF1A proteins: tumour cells promote cell growth and invasion by activating and maintaining membrane-based ion exchangers reversing the pH gradient across cell membrane (intracellular alkalinisation), thus causing the release of eEF1A proteins from actin filaments and their migration in different cellular compartments. Moreover, a weakened association of eEF1As to actin has been proposed to be related to cancer invasion (Edmonds *et al*, 1996). Thus, it is possible that the decrement of the eEF1A2 protein at cytoskeletal/nuclear fraction, as well as its distribution within the cells, could play an important role in promoting and sustaining aggressiveness.

Whereas functional studies about the eEF1As role in prostate cancer go below the scope of this work, we have conducted preliminary studies in this sense. We observed (data not shown) that the inhibition of eEF1As functions by an aptameric ssDNA molecule known to bind to cytoskeletal/nuclear fraction of eEF1As (Dapas *et al*, 2003; Scaggiante *et al*, 2006), reduced prostate tumour cell growth. The effect was dose dependent and the highest dose (0.25 *μ*M) inhibited cell growth by about 65% of controls; the effect was detectable up to 10 days after a single administration. This preliminary observation is in favour of a mechanical contribution of eEF1As to prostate cancer cell growth.

The cellular lines of prostate tumours tested in this work have been chosen to cover the vast majority of the different forms of prostate tumour occurring *in vivo*: the DU-145 and PC-3 resemble the less differentiated and aggressive prostate tumour, the LNCaP cells resemble the most differentiated and androgen-responsive prostate tumour forms and the 22Rv1 cells represent a cellular model of the progression towards hormonally refractory tumours. This is particularly relevant as hormone-refractory relapse is an inevitable and lethal event for advanced prostate carcinoma patients after hormone deprivation (Feldman and Feldman, 2001). Together, the data collected from these different models of prostate tumour *in vitro* strongly suggest that the eEF1A2 levels correlate with the transformation of prostate cancer cells. This observation is in agreement with the data we obtained from four Finefix-fixed paraffin-embedded (Figure 5A and B) and nine formalin-fixed (Figure 5D and E) human prostate cancer tissues; in almost all the hyperplastic peritumoural and in 10 out of 13 tumour tissue sections, but not in normal prostate tissue sample or in benign hyperplasia, EEF1A2 mRNA was detectable. In agreement with this observation, our preliminary quantification *in vivo* using prostate tissue microarrays (unpublished results) confirms eEF1A2 expression in tumour tissue sections. In contrast, EEF1A1 mRNA expression was detectable in all kinds of tissues analysed, once again in agreement with the cell lines data.

In conclusion, the results here presented support the concept that EEF1A2 switch-on is a feature of prostate cancer, assigning to EEF1A1 a minor involvement as potential marker of cancer. Whereas a higher number of prostate tissues need to be analysed, the data here presented are encouraging to consider eEF1A2 as an hallmark for prostate cell tumour transformation and may be progression. This opens the possibility to consider eEF1A2 content/distribution useful for a more defined prostate cancer diagnosis.
