# Supplementary figures and images for: Dissecting the expression of EEF1A1/2 genes in human prostate cancer cells: the potential of EEF1A2 as a hallmark for prostate transformation and progression
Source: Br J Cancer. 2011 Nov 17;106(1):166–73. doi: 10.1038/bjc.2011.500 (PMC3251850; doi:10.1038/bjc.2011.500)

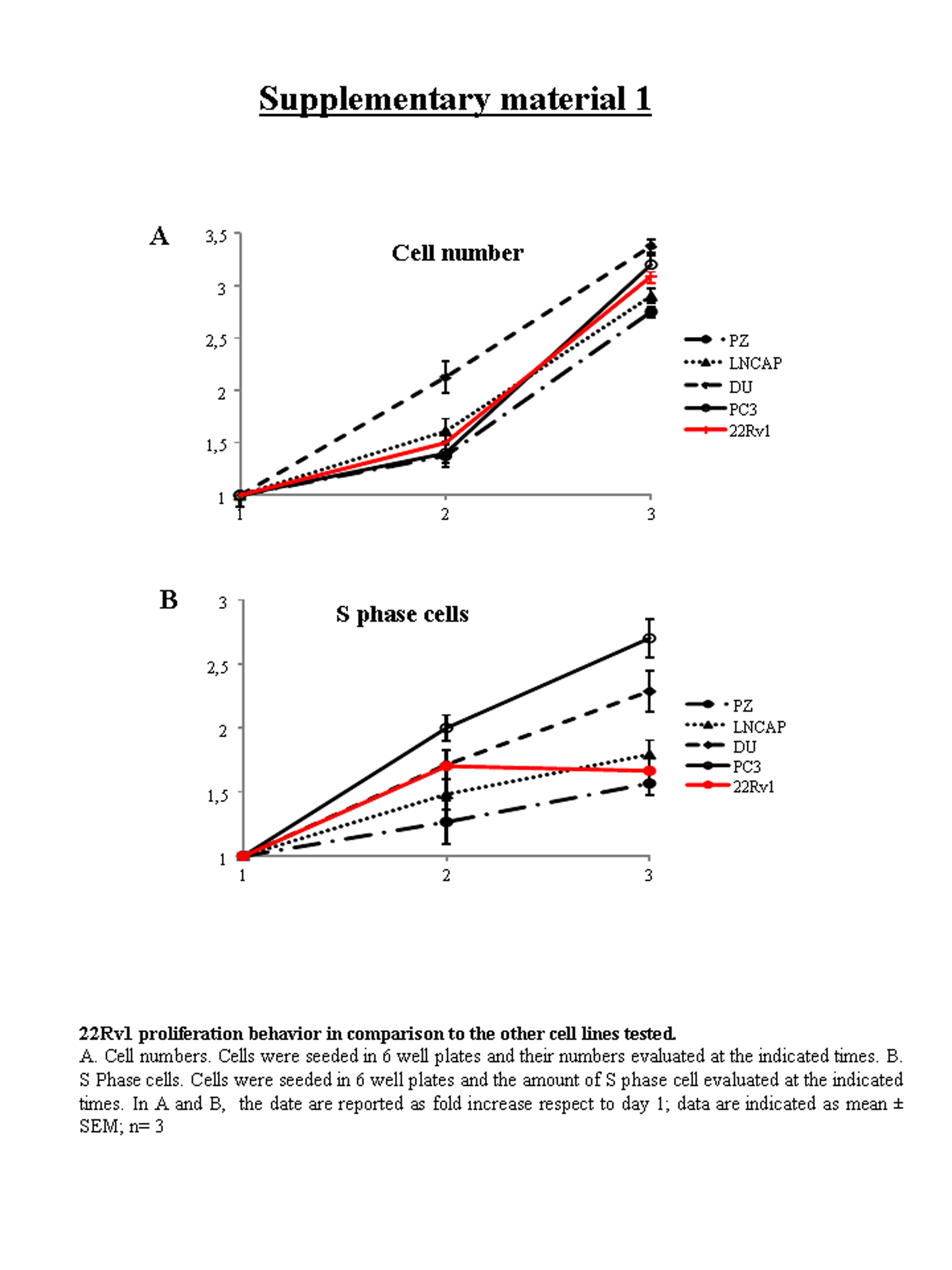

Supplement: Supplementary Material 1 [file bjc2011500x1.tif]

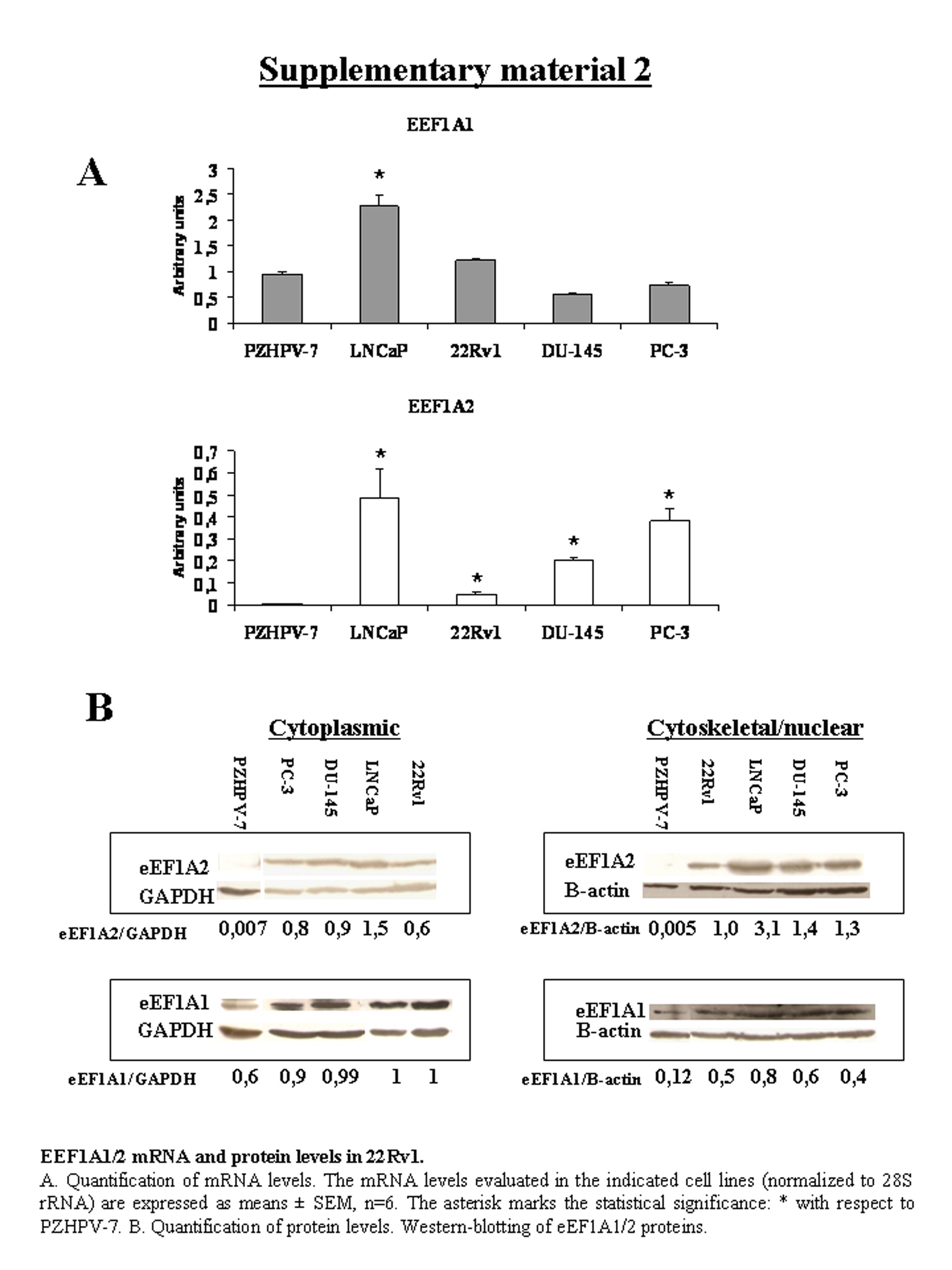

Supplement: Supplementary Material 2 [file bjc2011500x2.tif]
